# Rumen microbiota responses to the enzymatic hydrolyzed cottonseed peptide supplement under high-concentrate diet feeding process

**DOI:** 10.3389/fvets.2022.984634

**Published:** 2022-11-11

**Authors:** Peng Ma, Yifen Hong, Chunxue Liu, Yuqin Sun, Minze Liu, Zhengang Yang, Pengyun Ma, Hongxiang Wu, Fuguang Xue

**Affiliations:** ^1^Nanchang Key Laboratory of Animal Health and Safety Production, Jiangxi Agricultural University, Nanchang, China; ^2^Anyou Biotechnology Group Co. Ltd., Taicang, China; ^3^Yangxin Yiliyuan Halal Meat Co. Ltd., Yangxin, China

**Keywords:** small peptide, rumen, rumen microbiota, nutrient degradability, *in vitro* fermentation

## Abstract

In current dairy production, dietary energy is always excessively provided with a high-concentrate diet feeding to improve milk production. However, this feeding practice disturbed the rumen microbial ecosystem and the balance between ruminal energy and nitrogen, resulting in decreased nutrient fermentability, which in turn declined the milk yield of dairy cows. Therefore, supplementation of dietary degradable nitrogen may be helpful for high dairy production. In this study, we evaluated the regulatory effects of easily utilized enzymatic hydrolyzed cottonseed peptide (EHP) supplements on rumen microbiota communities and rumen nutrient fermentability under high-concentrate feeding. For this purpose, a gradient concentrate of EHP (from 0.2 to 1.0%) was added to the high-concentrate basal substrates for an i*n vitro* experiment. Each treatment contained three replicates, with three bottles in each replicate. Rumen fermentable parameters included microbial protein content, volatile fatty acids, and ammonia-N; the rumen nutrient degradability of dry matter, crude protein, neutral detergent fiber, acid detergent fiber, ether extracts, calcium, and phosphorus were further investigated after *in vitro* fermentation for 72 h. Then, rumen microbiota communities and their correlation with ruminal fermentation parameters and rumen nutritional degradability were analyzed to understand the regulatory mechanism of the EHP supplements on rumen fermentability. Results indicate that treatment with 0.6% of EHP supplements had the highest content of acetate, butyrate, and neutral detergent fiber degradability among all treatments. Furthermore, EHP supplements significantly increased the relative abundance of rumen cellulose and starch-degrading bacteria such as *Ruminococcus, Bifidobacterium*, and *Acetitomaculum*, and the high nitrogen utilizing bacteria *Butyrivibrio* and *Pseudobutyrivibrio*, which may further promote the rumen carbohydrate and nitrogen metabolism. In summary, supplementation of easily degraded small peptides helps reestablish rumen energy and nitrogen balance to promote the rumen fermentable functions and nutritional degradability under high-concentrate diet feeding circumstances. These findings may further promote dairy production.

## Introduction

The rumen is the basal nutrient-degradative organ to maintain ruminants' growth and normal physiological functions through equilibrated microbial utilization of dietary carbon and nitrogen sources (1, 2). The rumen energy and nitrogen balance (RENB) provide the preliminary basal environment for microbial proliferation and nutrient utilization (3, 4). Proliferated rumen microbiome retroactively consolidated the rumen micro-ecosystem homeostasis and enhanced the utilization efficiency of dietary nutritional substrates (5). However, energy-abundant high-concentrate diets are more preferentially offered to the lactating dairy cows in the modern dairy industry to achieve high milk production, and this feeding practice has been reported to disrupt the RENB, ruminal microbial homeostasis, and cause serious nutritional diseases such as ruminal subacute acidosis (6). Therefore, proper strategies to reestablish the RENB under high-concentrate dietary feeding procedures are critically required to promote dairy cows' production.

Generally, rumen-degradable proteins (RDPs) are the main easily acquired nitrogen sources for microbial proliferation and RENB maintenance. In the current feeding procedure, dairy cow diets contain a large amount of soybean meal and other protein-abundant feedstuffs; however, rumen degradable nitrogen (RDN) content is still lower than the requirement when considering its equilibrium with high dietary energy provision (7, 8). In addition to the lack of RDN, the proliferation of both cellulolytic and amylolytic bacteria was suppressed, which further decreased nutrient degradation and nutritional provision for dairy cows (9). Thus, supplementation of easily degraded nitrogen resources to efficiently increase RDN content is critical to maintaining the rumen functions.

Small peptides are the ideal resources for easily degradable nitrogen because they can be degraded by peptidases into AA and further utilized by rumen microbiota for their proliferation and production of microbial protein nitrogen (10). Subsequently, microbial proteins are transported into circulation and then utilized by dairy body tissue or organs (11, 12). Among rumen bacteria, *Peptostreptococcus anaerobius* and *Clostridium* are the main hyper-ammonia and microbial protein-producing genera (13). Notably, the abundances of these two genera decreased during high-concentrate feeding, which further highlighted the insufficient RDN supply and impaired bacterial growth and microbial protein production under high-concentrate conditions. Therefore, we hypothesized that supplementation of easily degradable small peptides in high-concentrate diets would improve RENB and nutritional availability by balancing the rumen microbiome. Specifically, 0.2–1.0% enzymatic hydrolyzed cottonseed peptides (EHP) were added to the high-concentrate diets, and the appropriate EHP supplement ratio was evaluated by ruminal fermentation parameters and nutritional degradability. In addition, we conducted metagenomic and correlation analysis to reveal the regulation of EHP on the rumen microbial community and to elucidate the possible mechanism of EHP on improved rumen fermentation and nutritional degradability.

## Materials and methods

Animal care and procedures in this study were conducted according to the Chinese Guidelines for Animal Welfare, which were approved by the Animal Care and Use Committee of Jiangxi Agricultural University (JXAULL-20220218).

### Experimental design

Rumen fluid was obtained from three rumen-cannulated Chinese Holstein dairy cows that were reared on the dairy cattle farm of the Jiangxi Agricultural University. All cows were fed in the same stall and fed the same diets during the trial period.

The *in vitro* experiments were conducted in a 75-ml fermentation system as reported by Ranilla (14). Fermentation substrates consisted of a 0.5-g feed sample, 50 ml of medium (pH, 6.86), and 24 ml of rumen fluid. Basal substrates were provided with a high-concentrate diet (HC; concentrate/forage, 6:4, CP, 18.10%, NDF, 27.61%, starch, 30.82%, DM basis), while EHP was added to the basal diet in a gradient from 0.2 to 1.0%. The ingredients and nutritional level of each treatment are listed in Table 1. Each treatment included 6 repeats, with 3 bottles in each repeat. The *in vitro* fermentation procedure lasted for 72 h each time and was repeated three times.

**Table 1:** Ingredients and chemical composition of the fermented substrates (dry matter basis).

**Items**	**HC**	**0.20%**	**0.40%**	**0.60%**	**0.80%**	**1.00%**
Ingredients (%)						
Corn silage	20.5	20.5	20.5	20.5	20.5	20.5
Ground Corn	19.7	19.7	19.7	19.7	19.7	19.7
Cottonseed meal	3.4	3.4	3.4	3.4	3.4	3.4
Alfalfa hay	10.5	10.5	10.5	10.5	10.5	10.5
Chinese Wildrye	10.2	10.2	10.2	10.2	10.2	10.2
Distillers Dried Grains With Solubles (DDGS)	3.1	3.1	3.1	3.1	3.1	3.1
Steam-flaked corn	12.5	12.5	12.5	12.5	12.5	12.5
Soybean meal	12	11.8	11.6	11.4	11.2	11
Small peptide	0	0.2	0.4	0.6	0.8	1
Beet pulp	4.5	4.5	4.5	4.5	4.5	4.5
Premix^*a*^)	3	3	3	3	3	3
NaCl	0.6	0.6	0.6	0.6	0.6	0.6
Total	100	100	100	100	100	100
Chemical composition (%)						
DM	51.2	51.2	51.2	51.2	51.2	51.2
NE (MJ/kg)	7.13	7.13	7.13	7.13	7.13	7.13
EE	4.56	4.56	4.56	4.56	4.56	4.56
CP	18.1	18.1	18.1	18.1	18.1	18.1
RDP	10.76	10.85	10.94	11.03	11.12	11.21
ADF	18.6	18.6	18.6	18.6	18.6	18.6
NDF	27.6	27.6	27.6	27.6	27.6	27.6
Starch	30.8	30.8	30.8	30.8	30.8	30.8
Ca	0.69	0.69	0.69	0.69	0.69	0.69
P	0.44	0.44	0.44	0.44	0.44	0.44

a The components contained in the premix are as follows: Fe, 1,400 mg; Cu, 1,200 mg; Mn, 2,400 mg; Zn, 5,500 mg; Se, 40 mg; Co, 30 mg; I, 90 mg, VA, 900,000 IU; VD, 700,000 IU; VE, 9,000 IU.

RDP was calculated using the equation RDP, A+B[Kd/(Kd+Kp)], where, A, non-protein nitrogens and soluble proteins; B, potentially degradable proteins; Kd, rumen digestibility of B; Kp, velocity of circulation in the rumen.

### Rumen degradability measurement

Substrate samples were first collected within each treatment and mixed well, then dried at 65°C for further nutrient degradability analysis, including the dry matter (DM), organic matter (OM), crude protein (CP), neutral detergent fiber (NDF), and acid detergent fiber (ADF). Dry matter content was analyzed according to the AOAC Official Method 930.15 (15), and OM content was measured using the following equation:

OM% (DM basis), 100%-ash%.

Crude protein content was measured according to Qiu et al. (16) and Xia et al. (17), while NDF and ADF contents were analyzed according to Koch et al. (18). Briefly, 1 g of the sample was first placed into 100 ml of a neutral detergent (ND) with 50 μl of heat-stable amylase (dietary fiber kit; Sigma catalog number A3306), followed by placing the container on heat. Finally, Ankom 200 Fiber Analyzer (Ankom Technology, Fairport, NY) was used for the analysis of NDF content. For the ADF measurement, diets and fermentable residue were first treated with permanganate, and then the ratio of residue to the original content was calculated.

### Microbial community measurement

The microbial community was measured according to the method described by Xue et al. (19) and Chen et al. (20). To be simply stated, rumen fluid DNA was first extracted using Bacterial Genome DNA Extraction Kit (DP302, TIANGEN, TIANGEN BIOTECH (BEIJING) Co., Ltd). Furthermore, the V4 region of 16S rRNA was amplified by the primers 520F and 802R (F: GTGCCAGCMGCCGCGGTAA and R: GGACTACHVGGGTWTCTAAT). Qiagen Gel Extraction Kit (Qiagen, Hilden, Germany) was used to purify the mixture of PCR products. Then, sequencing libraries were generated using TruSeq^®^ DNA PCR-Free Sample Preparation Kit (Illumina, USA). Library quality was assessed through the Qubit^@^ 2.0 Fluorometer (Thermo Scientific) and Agilent Bioanalyzer 2100 systems, and finally, Illumina HiSeq 4000 platform (Illumina Inc., San Diego, USA) was applied for the sequencing process. Quality filtering of raw tags was performed under specific filtering conditions to obtain high-quality clean tags according to the Quantitative Insights Into Microbial Ecology (QIIME, Version 1.7.0) quality control process. Sequences with similarity showed that >97% were assigned to the same operational taxonomic unit (OTU).

### Statistical analysis

Ruminal fermentable parameters and rumen degradability-related parameters were first subjected to a normal distribution test using the SAS procedure “proc univariate data, test normal” and subsequently analyzed using the one-way ANOVA S-N-K test of SAS 9.2 (SAS Institute, Inc., Cary, NC, USA). Significance would be considered when *P* < 0.05, while a tendency was considered when 0.05 ≤ *P* < 0.10. OTU abundances of each rumen bacteria were first conducted through a percentage transformation, followed by a one-way ANOVA S-N-K test of SAS 9.2. Alpha diversity and beta diversity in our samples were calculated using QIIME 2 (21) and displayed with R software (Version 3.3.1, R Core Team, Vienna, Austria). Principal coordinate analysis (PCoA) for different rumen communities was conducted using the R “vegan package.” Spearman correlation analysis between fermentability parameters, degradability parameters, and bacteria communities was assessed using the PROC CORR procedure of SAS 9.2 to create a correlation matrix and then visualized in a heatmap format using R software (Version 3.3.1).

## Results

### EHP supplements in a high-concentrate diet improved rumen fermentation *in vitro*

To evaluate the beneficial effects of EHP on rumen function during high-concentrate feeding, we first determined the rumen fermentation parameters, including rumen microbial protein, rumen ammonia-N, VFAs, and gas production *in vitro*. As shown in Table 2, compared with HC treatment, supplementation of 0.2% and 0.6% EHP effectively increased the rumen ammonia-N content while supplementation of 0.8 and 1.0% EHP significantly increased microbial protein generation (*P* < 0.05). In terms of VFAs, all the EHP content supplementation significantly increased the acetate/propionate ratio compared with HC treatment (*P* < 0.05). Specifically speaking, supplementation of 0.6% EHP significantly increased the levels of acetate and butyrate content compared with the HC group (*P* < 0.05); however, no significant differences in propionate content were observed. Besides, supplementation of 0.2, 0.8, and 1.0% EHP significantly decreased ruminal propionate content compared with CON (*P* < 0.05). No other significant discrepancies were observed among all treatments. For the total gas production, no significant changes were observed among all treatments.

**Table 2:** Effects of small peptide supplement in high-concentrate diets on rumen fermentation parameters (*n*, 6).

**Items**	**Experimental treatments**						**SEM**	***P*-value**
	**HC**	**0.20%**	**0.40%**	**0.60%**	**0.80%**	**1.0%**		
Ruminal pH	5.97	6.02	6.01	6.02	5.99	6.08	0.094	0.216
MCP (mg dL-1)	22.16^b^	25.99^a^	23.23^b^	26.34^a^	23.74^b^	21.57^b^	1.741	0.43
Ammonia-N (mg/100 mL)	11.49^b^	12.41^ab^	12.54^ab^	12.77^ab^	13.96^a^	13.27^a^	0.981	0.006
Acetate (mmol/L)	44.24^b^	44.85^b^	46.26^ab^	47.27^a^	45.28^b^	44.07^b^	1.273	0.038
Propionate (mmol/L)	12.49^a^	11.38^b^	12.11^a^	12.13^a^	11.75^b^	11.44^b^	0.632	0.027
Acetate/Propionate	3.54^b^	3.94^a^	3.82^a^	3.90^a^	3.85^a^	3.85^a^	0.113	0.043
Butyrate (mmol/L)	10.77^b^	11.12^b^	12.28^a^	12.75^a^	11.33^b^	12.01^ab^	0.37	0.356
Total gas production (mL·g-1 DM)	116.7	113.4	112.3	118.4	116.4	115.2	5.484	0.316

a,b means within a row with different letters differed significantly (*P* < 0.05); SEM, standard error of the mean.

HC, high-concentrate diet; MCP, microbial protein.

### EHP supplement in a high-concentrate diet enhanced rumen nutrient degradability *in vitro*

Rumen VFAs, ammonia-N, and microbial protein are the main microbial productions from dietary proteins and fibers. Thereafter, we detected the effect of the EHP supplement on nutrient degradability under high-concentrate conditions. All results are shown in Table 3. Supplementation of 0.40, 0.60, 0.80, and 1.0% of EHP significantly enhanced NDF degradability when compared with HC treatment (*P* < 0.05). Meanwhile, supplementation with 0.60, 0.80, and 1.0% of EHP significantly promoted CP degradation compared with the other treatments (*P* < 0.05). No significant differences were detected for the degradability of DM, ADF, EE, Ca, and P. These results, together with rumen fermentation parameters, demonstrated that an EHP supplement at a dosage of 0.60% was enough to improve rumen fermentation and nutrient degradability.

**Table 3:** Effects of small peptide supplement in high-concentrate diets on rumen nutritional degradability (*n*, 6).

**Items (%)**	**Experimental treatments**						**SEM**	***P*-value**
	**HC**	**0.20%**	**0.40%**	**0.60%**	**0.80%**	**1.00%**		
DM	58.66	59.21	59.46	60.23	59.64	58.31	1.094	0.216
NDF	62.64^b^	63.88^ab^	64.34^a^	64.97^a^	64.85^a^	64.21^a^	1.141	0.043
ADF	57.62	57.33	57.89	58.21	58.13	57.36	1.273	0.338
EE	67.23	67.31	66.29	66.88	68.21	66.41	3.632	0.527
CP	61.37^b^	61.76^b^	62.23 ^b^	63.12 ^a^	63.34 ^a^	63.27 ^a^	1.113	0.043
Ca	45.09	45.12	44.78	44.96	45.21	45.06	3.371	0.356
P	41.32	40.21	41.37	42.01	42.64	41.63	2.484	0.316

a,b means within a row with different letters differed significantly (*P* < 0.05); SEM, standard error of the mean.

HC, high-concentrate diet; DM, dry matter; NDF, neutral detergent fiber, ADF, acid detergent fiber; EE, ether extracts; CP, crude protein; Ca, calcium; P, phosphorus.

### EHP supplement in high-concentrate diet modulated rumen microbial communities

To elucidate the underlying mechanism of EHP on improved rumen fermentation and nutritional degradability, we conducted metagenomic analysis within HC and 0.60% EHP supplement treatments to analyze the change in rumen microbial community composition in response to EHP supplement. In general, a total of 4,213 OTUs were obtained according to 97% similarity. A total of 15 phyla and 2,134 genera were identified after quality control and taxonomic analysis. All the taxonomic information is displayed in Supplementary Table S1. Thereafter, all taxonomic bacteria were applied for α-diversity, β-diversity, and differential communities' investigation.

### Diversity of rumen bacterial microbiota in response to EHP supplement

#### α-diversity

Alpha diversity was first applied to determine the complexity of rumen microbial diversity through the Chao1, Shannon, Simpson, observed species, and Ace indexes. Based on the results in Table 4, alpha diversity indexes including Chao1, Shannon, observed-species, and Ace indexes are all significantly increased after EHP supplement treatment (*P* < 0.05) compared with HC. No discrepancy was observed on the Simpson index after the EHP supplement compared with HC.

**Table 4:** Comparison of bacterial alpha diversity indices between the HC group and 0.60% EHP supplement (*n*, 6).

**Items**	**Experimental treatments**		**SE**	***P-*value**
	**HC**	**EHP**		
Chao 1	2324	2548	47.43	0.003
Ace	2325.2	2492.6	38.04	0.021
Observed-species	1802	1953	34.98	0.022
Shannon	7.35	8.20	0.18	0.018
Simpson	0.97	0.98	0.015	0.175

EHP, enzymatic hydrolyzed cottonseed peptide supplement treatment; HC, high-concentrate diets. SE, standard error.

#### β-diversity

**Figure 1:**
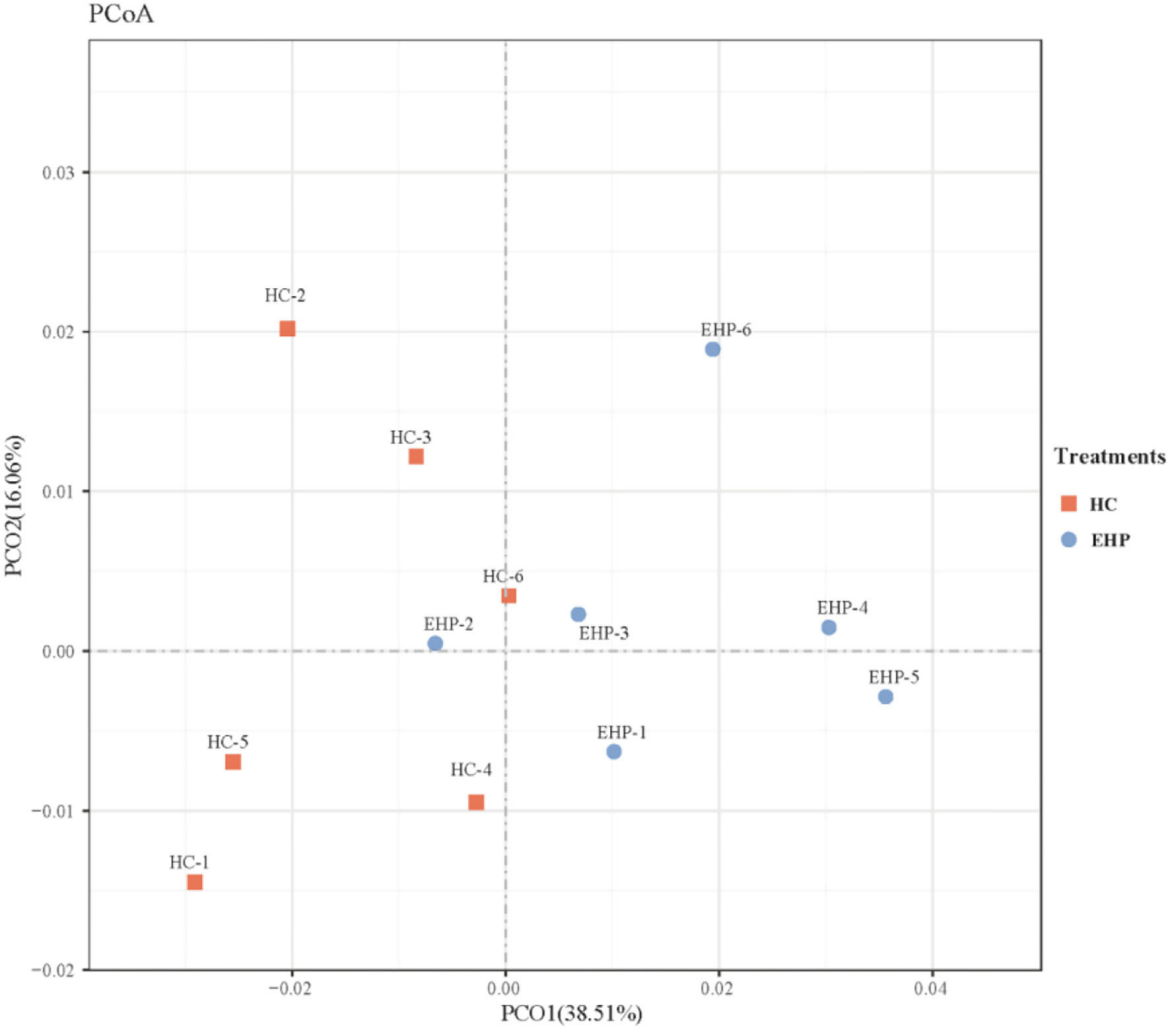
Principal coordinate analysis (PCoA) on community structures of the rumen microbiota between small peptide supplement and high-concentrate diet treatments. HC, high-concentrate diets; EHP, enzymatic hydrolyzed cottonseed peptides.

In terms of β diversity of the bacterial communities between two treatments, the PCoA analysis was conducted and illustrated based on unweighted Unifrac distances (Figure 1). As shown in Figure 1, PCoA axes 1 and 2 accounted for 38.51 and 16.06%, respectively. Bacterial communities in EHP treatment could be significantly separated from those in HC through PCoA axes 1 and 2, except in EHP2. These data indicated that EHP supplements distinctly increased rumen bacterial communities.

Differential analysis on the detailed relative abundance of the bacterial community was applied based on the OTU taxonomy results. The differential results are shown in Tables 5, 6.

**Table 5:** Effects of small peptide supplement in high-concentrate diets on relative abundances of ruminal bacterial communities (%) (level of phyla) (*n*, 6).

**Items**	**Experimental treatments**		**SE**	***P*-value**
	**HC**	**EHP**		
Bacteroidetes	44.78	42.51	1.274	0.041
Firmicutes	36.26	37.57	0.935	0.031
Proteobacteria	13.06	14.83	1.769	0.169
Fibrobacteres	0.99	1.64	0.058	0.028
Spirochaetes	0.95	1.04	0.166	0.187
Actinobacteria	1.42	0.71	0.044	0.008
Verrucomicrobia	0.32	0.34	0.031	0.07
Chlamydiae	0.44	0.59	0.059	0.101
Cyanobacteria	0.19	0.14	0.056	0.081
Tenericutes	0.14	0.16	0.017	0.301
Fusobacteria	0.07	0.12	0.011	0.158
others	1.38	0.35	0.016	0.002

EHP, enzymatic hydrolyzed cottonseed peptide supplement treatment; HC, high-concentrate diets; SE, standard error.

**Table 6:** Effects of small peptide supplement in high-concentrate diets on relative abundances of ruminal bacterial communities (%) (level of genera) (*n*, 6).

**Items**	**Experimental treatments**		**SE**	***P*-value**
	**HC**	**EHP**		
g__Prevotella	16.32	18.34	2.39	0.064
g__Ruminococcaceae	15.84	14.7	1.09	0.105
g__Succiniclasticum	9.89	10.21	0.82	0.291
g__Lachnospiraceae	5.22	5.16	0.28	0.623
g__Eubacterium	4.84	3.97	0.21	0.141
g__Rikenellaceae	2.77	2.14	0.25	0.015
g__Ruminococcus	5.01	7.31	1.88	0.012
g__Shuttleworthia	2.6	1.34	0.52	0.032
g__Prevotellaceae	1.27	1.22	0.23	0.371
g__Acetitomaculum	0.73	1.28	0.33	0.023
g__Erysipelotrichaceae	1.11	0.86	0.33	0.137
g__Lachnoclostridium	0.66	0.73	0.22	0.995
g__Saccharofermentans	0.63	0.45	0.11	0.008
g__Butyrivibrio	0.48	0.58	0.05	0.048
g__Ruminiclostridium	0.21	0.28	0.08	0.503
g__Lachnospira	0.32	0.16	0.07	0.034
g__Pseudobutyrivibrio	0.11	0.18	0.03	0.009
g__Acidaminococcus	0.13	0.07	0.07	0.167
g__Selenomonas	0.04	0.11	0.04	0.001
g__Lactobacillus	0.05	0.06	0.05	0.936
g__Pseudoramibacter	0.04	0.02	0.03	0.089
g__Bifidobacterium	0.03	0.05	0.01	0.027
g__Escherichia-Shigella	0.01	0.01	0.003	0.68
g__Bacteroides	0.01	0.02	0.01	0.571
g__Succinivibrio	0.03	0.01	0.003	0.001
g__Streptococcus	0.01	0.01	0.006	0.083
g__Butyricicoccus	0.01	0.01	0.007	0.969
Others	31.63	30.72	1.45	0.211

EHP, enzymatic hydrolyzed cottonseed peptide supplement treatment; HC, high-concentrate diets; SE, standard error.

*Bacteroidetes, Firmicutes*, and *Proteobacteria* contributed to the most abundant three bacterial communities, which occupied more than 95% of total bacteria. *Bacteroidetes* accounted for the largest proportion among all rumen bacteria but significantly decreased after EHP treatment (*P* < 0.05). Meanwhile, the second abundant bacteria *Firmicutes* significantly increased after EHP treatment (*P* < 0.05). Particularly, the relative abundance of *Fibrobacteres* significantly proliferated after EHP treatment compared with HC (*P* < 0.05), while *Actinobacteria* significantly decreased after EHP treatment (*P* < 0.05). No significant differences were observed for other bacterial phyla between HC and EHP.

Discrepancies in the relative abundances of bacterial genera were calculated, and the results are shown in Table 6. *Prevotella, Ruminococcaceae*, and *Succiniclasticum* contributed to the most abundant 3 genera and accounted for more than 40% of total microbiota profiles, while no significant discrepancy was observed for the above-mentioned top 3 genera between EHP and HC. *Ruminococcus, Butyrivibrio, Acetitomaculum, Pseudobutyrivibrio, Selenomonas, and Bifidobacterium* were significantly proliferated after EHP supplement treatment compared with HC (*P* < 0.05). Meanwhile, relative abundances of *Shuttleworthia, Saccharofermentans, and Succinivibrio* significantly declined in EHP treatment compared with HC (*P* < 0.05). No significant changes were found for other bacterial genera between EHP supplement treatment and HC treatment.

### Correlations between rumen fermentability and bacterial communities

**Figure 2:**
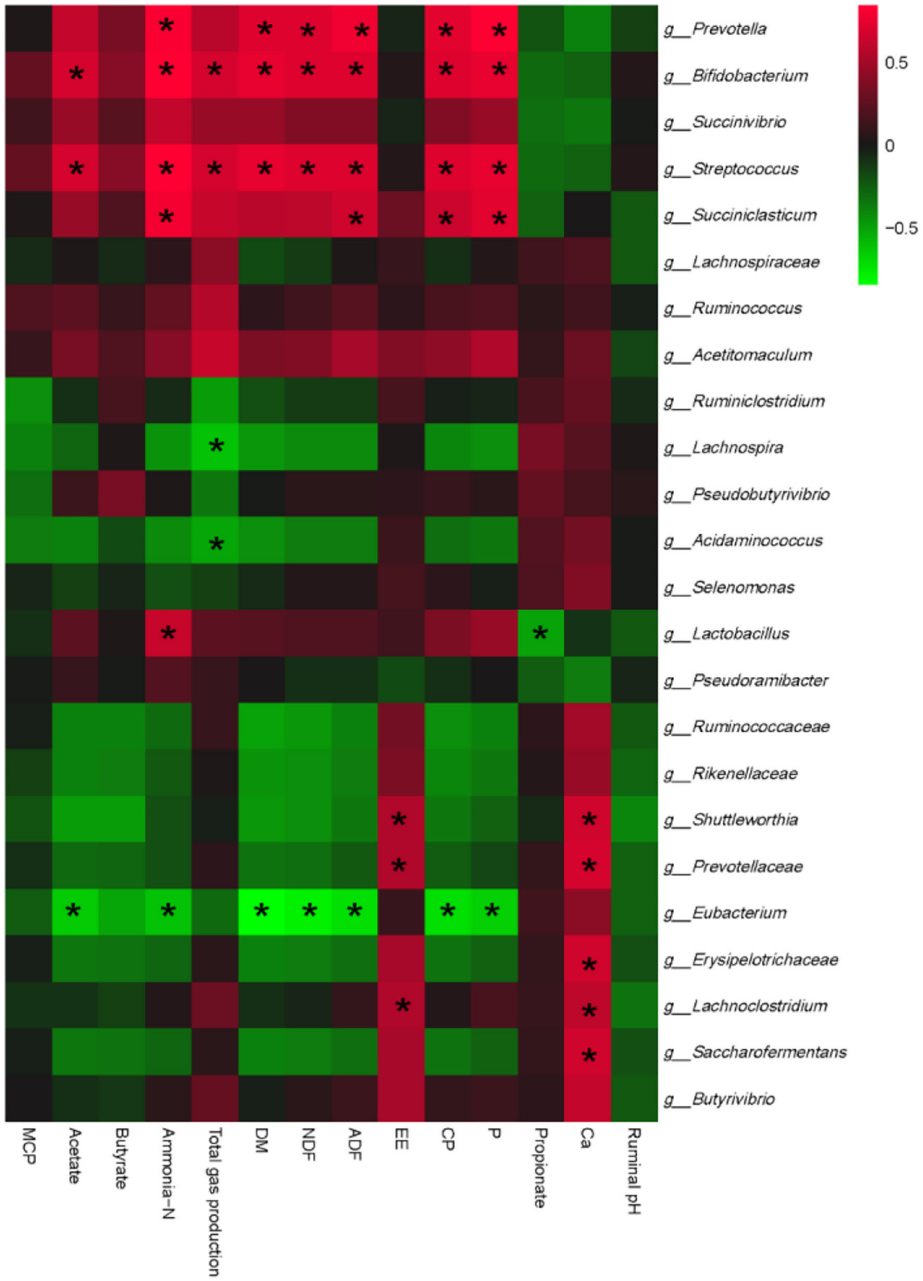
Correlation analyses between ruminal fermentation parameters, rumen nutritional degradability, and relative abundances of ruminal bacteria at the genus level. The red color represents a positive correlation, while the green color represents a negative correlation. “*” means a significant correlation (|r| > 0.55, *P* < 0.05). DM, dry matter; NDF, neutral detergent fiber; ADF, acid detergent fiber; EE, ether extracts; CP, crude protein; MCP, microbial protein.

To further clarify whether EHP-mediated rumen fermentation and nutrient degradability improvement are rumen bacteria dependent, we finally conducted an interactive analysis between ruminal fermentation parameters, ruminal nutrient degradability, and bacterial communities. As shown in Figure 2, bacterial communities could be separated into 2 big clusters. One cluster mainly consisted of *Prevotella, Bifidobacterium*, and *Streptococcus*, which showed a positive correlation with DM, NDF, CP, ADF degradability, and ammonia-N production, and negatively correlated with propionate content. The other cluster mainly consisted of *Eubacterium*, which showed a complete reverse correlation compared with the former one. Specifically, probiotics such as *Bifidobacterium* and *Lactobacillus* showed significant positive correlations with ammonia-N levels, while *Lactobacillus* showed a significant negative correlation with propionate. *Succiniclasticum* CP, P, ADF, and ammonia-N. *Bifidobacterium* and *Streptococcus* showed a positive correlation, while *Lachnospira* and *Acidaminococcus* showed a negative correlation with gas production. No other significant correlations were observed.

## Discussion

In ruminal conditions, the RENB provided the critical circumstance for both nutritional element supply and microbial autologous proliferation (21, 22). Under the circumstances, the rumen microbiome utilized carbohydrates and nitrogen to synthesize the ruminal microbial protein and VFAs for whole physiological function achievement and body growth. The optimum concentration of ruminal nitrogen appeared to be diet-dependent and influenced by factors such as types of nitrogen supplements, carbohydrate fermentability, and possible factors that affect passage rates (23, 24). Therefore, in this study, we investigated the regulatory effects of the enzymatic small peptide supplement on ruminal carbohydrate and nitrogen utilization, purposely to determine the potential application effects of EHP on ruminant production.

### Effects of small peptides on rumen carbohydrate fermentability

Dietary carbohydrates, which mainly included cellulose and starch, were degraded into acetate, propionate, and butyrate in ruminal conditions to provide the essential elements and energy for the growth and metabolism of ruminants. In our study, ruminal TVFA content, particularly the acetate and butyrate, significantly increased after 0.6% of small peptide supplementation. This result was in line with the findings of Valkeners et al. (5), who found that EHP supplements significantly increased carbohydrate fermentability. The main causative factor that increased VFA content should be attributed to the increased bacterial diversity after the EHP supplement. As an easily degradable and utilizing nitrogen source, small peptides provided certain degradable nitrogen for microbial proliferation and diversity enhancement, which could be reflected by the increment in α-diversity after EHP treatment (25). The increased ruminal bacterial communities further strengthen the microbial degradability, and thereafter, more carbon could be degraded into easily utilized VFAs for both self-development and nutrient provision.

Besides, the provision of more easily degradable proteins helps increase the relative abundances of *Firmicutes* and *Proteobacteria* under the high-concentrate diet feeding process (26, 27). These changes may trigger the active degradability of high-fermentable carbohydrates and lead to the increment of carbohydrate degradation into VFAs. In addition, despite the lower content of fiber provision, the degradation of cellulose played a critical role in physiological nutrient provision. Although the main fiber-degrading bacterial content such as *Bacteroidetes* and *fibrobacter* was significantly lower in this study compared with the previous, *fibrobacter* abundance was significantly higher after the EHP supplement, which may further promote the cellulose degradation and contribute to the increment of acetate and butyrate.

Moreover, VFAs, especially acetate, provide the primary resources for physiological bioactive substrate synthesis, which benefits ruminant development greatly. Acetate-generated bacteria such as *Acetitomaculum, Bifidobacterium*, and *Succinivibrio* significantly increased after the EHP supplement (28, 29). These increments in acetate-synthesizing bacteria may further promote carbohydrate degradation and contribute to the enhancement of acetate content.

### Effects of small peptides on rumen nitrogen fermentability

Apart from the carbohydrate metabolism, the average rumen NH_3_-N and MCP content after the EHP supplement significantly increased, which may in turn explain the effect of increased ruminal carbohydrate fermentability.

The NH_3_-N concentration in the rumen fluid reflected the equilibrium between the rumen-degrading rate of nitrogen in the diet and the rate of NH_3_-N utilization by the microorganisms (30). This parameter could be influenced by many factors such as solubility of feed protein, absorption of rumen wall, ruminal outflow rate, and rumen microbial community (31). As ruminal NH_3_-N concentration has been well reported to be positively correlated with rumen degradable protein content, supplementation with small peptides helps increase rumen degradable protein and therefore helps increase the NH_3_-N concentration. Furthermore, NH_3_-N was utilized by ruminal bacteria to form amino acids and was further synthesized into bacterial proteins in ruminal conditions. The increased MCP content in EHP supplement treatment might be attributed to the increased rumen RDP content after EHP supplement, which helps generate more MCP because the MCP content has been well reported to be positively correlated with rumen degradable protein and NH_3_-N concentration.

In addition, the utilization of ruminal NH_3_-N into the bacterial protein in the rumen is energy-dependent. Therefore, the provision of adequate ruminal available energy is positively correlated with ruminal NH_3_-N utilization (31, 32). The increased acetate and other VFA content in EHP treatment contributed to the energy provision, and therefore, more MCP could be synthesized and might further promote physiological function achievement and production.

In summary, supplementation with easily degraded small peptides helps promote the rumen fermentable functions and nutritional degradability under high-concentrate diet feeding. This finding may further theoretically promote ruminant production.

## Data availability statement

The datasets presented in this study can be found in online repositories. The names of the repository/repositories and accession number(s) can be found below: https://www.ncbi.nlm.nih.gov/, PRJNA867506.

## Ethics statement

The animal study was reviewed and approved by Animal Care and procedures followed the Chinese Guidelines for Animal Welfare, which was approved by the Animal Care and Use Committee of Jiangxi Agricultural University, with the approval number JXAULL-20220218.

## Author contributions

Conceptualization: FX and HW. Methodology and writing—reviewing and editing: FX. Validation: PengM and YH. Formal analysis and data curation: YS. Investigation: PengM, PengyM, ML, and ZY. Resources: ZY. Writing—original draft preparation: PengM. All authors have read and agreed to the published version of the manuscript.

## Funding

This research was supported by the Science and Technology Planning Project of the Jiangxi Educational Department (GJJ200414) and the Latitudinal Project of Jiangxi Agricultural University (2021JXAUHX021).

## Conflict of interest

Authors PengM, YH, and CL were employed by Anyou Biotechnology Group Co. Ltd., China. Authors YS, ML, and ZY were employed by Yangxin Yiliyuan Halal Meat Co. Ltd. The remaining authors declare that the research was conducted in the absence of any commercial or financial relationships that could be construed as a potential conflict of interest.

## Publisher's note

All claims expressed in this article are solely those of the authors and do not necessarily represent those of their affiliated organizations, or those of the publisher, the editors and the reviewers. Any product that may be evaluated in this article, or claim that may be made by its manufacturer, is not guaranteed or endorsed by the publisher.

## Supplementary material

Supplementary Table S1 Taxonomy results of each sample of both EHP and CON treatments.
